# A Nonlinear Association between Tongue Fur Thickness and Tumor Marker Abnormality: A Cross-Sectional Study

**DOI:** 10.1155/2021/7909850

**Published:** 2021-11-30

**Authors:** Ke Zhu, Yongsong Guo, Zhaohui Tang, Jian He, Bing Lin, Weihong Li

**Affiliations:** ^1^Basic Medical College, Chengdu University of Traditional Chinese Medicine, Chengdu, China; ^2^Nuclear Medicine Department, Affiliated Hospital of Chengdu University of Traditional Chinese Medicine, Chengdu, China; ^3^Health Management Center, Affiliated Hospital of Chengdu University of Traditional Chinese Medicine, Chengdu, China

## Abstract

**Background:**

Many associations between tongue fur and different physiological and biochemical indexes have been revealed. However, the relationship between tongue fur and tumor markers remains unexplored.

**Methods:**

We collected the medical examination reports of 1625 participants. Participants were residents of Chengdu, China, undergoing routine health checkups at the health management center of the Affiliated Hospital of Chengdu University of Traditional Chinese Medicine between December 2018 and September 2020. The participants' tongue fur thickness was measured using the DAOSH four-diagnostic instrument. Tumor marker levels, including t-PSA, AFP, CEA, CA125, and CA199, were measured in the clinical laboratory. Curve-fitting and multivariable logistic regression were used to analyze the association between tongue fur thickness and tumor marker abnormality.

**Results:**

Curve-fitting showed that the relationship between tongue fur thickness and abnormal tumor marker rate was nonlinear, similar to a U shape. As the tongue fur thickness value increased, the abnormal tumor marker probability initially decreased and then increased. Logistic regression showed that, in the crude model, compared with the thin tongue fur group, the odds ratios (ORs) and 95% confidence intervals (CIs) of the less or peeling tongue fur group and thick tongue fur group for tumor marker abnormality were 1.79 (1.02–3.17) and 1.70 (1.13–2.54), respectively. After adjusting gender, age, body mass index (BMI), smoking history, drinking history, tongue color, the form of the tongue, and fur color, the ORs and 95% CIs of the less or peeling tongue fur group and thick tongue fur group were 1.93 (1.04–3.57) and 1.82 (1.17–2.81), respectively.

**Conclusions:**

Excessive or very little tongue fur is associated with tumor marker abnormality. Further cross-sectional studies are needed to evaluate the clinical value of tongue fur for cancer diagnosis and screening.

## 1. Introduction

Tongue diagnosis has been widely used in both traditional Chinese medicine (TCM) and ancient Greek medicine [1, 2]. Tongue fur (also called tongue coating) examination, as an essential procedure of tongue diagnosis, still plays a role in TCM daily practice to date. According to TCM theory, stomach qi, a vital life force, nourishes all five viscera and six bowels and helps generate tongue fur. Given this connection, tongue fur manifestation is regarded as a mirror of the internal organs' state by TCM practitioners [3].

Modern medical research has uncovered the essence of tongue fur. It consists of desquamated epithelial cells, blood cells, metabolites, nutrients, and bacteria [4]. Many factors can affect the formation of tongue fur which are as follows: (1) the entrapment of food particles, saliva, and bacteria in-between filiform papillae; (2) the balance between retaining and removing keratinized and nonkeratinized epithelial cells; and (3) the rates of epithelial cell multiplication and membrane coating granule production [5].

Interestingly, in line with the TCM view, scientific research also discovered the connection between tongue fur and inner health condition. For instance, yellow tongue fur is reported to be associated with a higher prevalence of insulin resistance and diabetes [6, 7], and thick tongue fur is identified to be associated with lower low-density lipoprotein (LDL) [8], higher high-density lipoprotein (HDL) [9], and higher neutrophil count [10]. Although many associations of tongue fur with different physiological and biochemical indexes have been revealed, the relationship between tongue fur and tumor markers remains unexplored.

With data mining techniques, we found a nonlinear association between tongue fur thickness and serum tumor marker abnormality using data from a study titled “A Real-World Study for the Medical Data of Four Diagnostic Synergies Centered on Tongue Image Data for Major Diseases” (ChiCTR1800018090, data unpublished) [11]. In this study, we aimed to further confirm this association by setting narrower exclusion criteria and adjusting potential confounders in multivariable regression models.

## 2. Methods

### 2.1. Study Design

The study design is cross-sectional. Participants included in this study were residents living in Chengdu, China, undergoing routine health checkups at the health management center of the Affiliated Hospital of Chengdu University of TCM between December 2018 and September 2020. Most participants only attended one health checkup during the study period. For the few participants who attended health checkups more than once, we only collected their first health reports for data analysis. The routine health checkup includes anthropometric and basic clinical assessment (weight, height, and blood pressure), biochemical tests (such as blood routine examination, hepatic function, renal function, and trace elements test), and imaging tests (such as chest radiography and abdominal ultrasonography).

### 2.2. Eligibility Criteria

**Figure 1:**
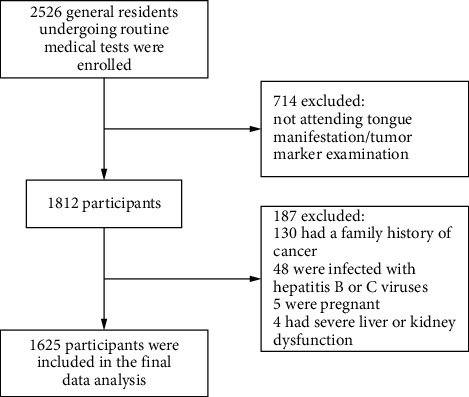
The flowchart of the study sample selection.

Inclusion criteria were as follows: (1) over 18 years old; (2) attending the TCM intelligent tongue image examination willingly; and (3) attending the detection of serum tumor markers willingly. Exclusion criteria were as follows: (1) with a family history of cancer; (2) infected with hepatitis B or C virus; (3) severe liver and kidney dysfunction; and (4) pregnant women. After screening, 1,625 qualified participants were included in the study (Figure 1). The Ethics Committee of the Affiliated Hospital of Chengdu University of TCM approved this study (2018-KL050), and all participants provided written informed consent.

### 2.3. Assessment of Tongue Fur Thickness

In the morning, the participants were on an empty stomach. After gargling, the participants placed their heads in a DAOSH four-diagnostic instrument (Shanghai Food & Drug Administration approval no. 20202200060 for medical devices). Under the standard light source, the tongue images were taken and automatically determined by the DS01-A tongue, complexion, pulse, and constitution information acquisition and identification system (Daosh Co., Shanghai, China). The density of tongue fur distribution and the approximate degree of fur color and tongue color were used to describe the tongue fur's depth, that is, the thickness degree [12]. Based on the thickness degree, the tongue fur can be divided into three categories: thin fur, less or peeling fur, and thick fur. The mathematical formula of tongue fur thickness degree is as follows:

(1) $$\begin{array}{c} cf\left(i\right)=-\frac{1}{m}\left(\sum_{i=1}^{m}\left(\alpha_{1}\times cr_{i}+\alpha_{2}\times ep_{i}+\alpha_{3}\times cd_{i}\right)\right), \end{array}$$

where *cf*(*i*) is tongue fur thickness degree. *cr*_*i*_ is the coverage ratio of tongue fur. *ep*_*i*_ is the exposure of the tongue under the tongue fur. *cd*_*i*_ is the color difference between tongue fur and tongue nature. *α*_1_, *α*_2_, *α*_3_ are correlation coefficient. The tongue target image is divided into *m* regions. On the *m* regions, the parameters *α*_1_, *α*_2_, *α*_3_, about coverage ratio of tongue fur, the exposure of the tongue under the tongue fur, the color difference between tongue fur and tongue nature in the abovementioned formula are adjusted, according to the results of *cf*(*i*) and thresholds classify the tongue fur as thick or thin [13].

### 2.4. Measurement of Tumor Markers

Participants' peripheral venous blood was extracted at a fasting state, and then the tumor markers t-PSA, AFP, CEA, CA125, and CA199 were measured in the Nuclear Medicine Department of the Affiliated Hospital of Chengdu University of TCM. All the tumor markers were determined by electrochemiluminescence immunoassay. The detection instrument was Cobas 6000 e601 (Roche, Switzerland), and the detection reagent was purchased from Roche Diagnostics GmbH (Mannheim, Germany).

### 2.5. Collection of Other Covariates

Other collected covariates included age, gender, height, weight, smoking history, drinking history, tongue color, the form of the tongue, and fur color. Participants' smoking and drinking histories were inquired by experienced clinicians. Height and weight were measured using standardized electronic instruments. Tongue color, the form of the tongue, and fur color were determined using the DAOSH four-diagnostic instrument.

### 2.6. Statistical Analysis

Continuous variables were expressed as mean ± standard deviation (normal distribution) or median (quartile) (skewed distribution), and categorical variables were expressed in frequency or as a percentage. The one-way ANOVA (normal distribution), the Kruskal–Wallis H test (skewed distribution), and the chi-square test (categorical variables) were used to determine any statistical differences between the means and proportions of the groups. When addressing tongue fur thickness as a continuous variable, smooth curve-fitting was used to describe the variation trend of abnormal tumor marker rate with tongue fur thickness. When tongue fur thickness was analyzed as a categorical variable, multivariable logistic regression was used to evaluate the association between tongue fur thickness and tumor marker abnormality. Both nonadjusted and multivariable-adjusted models were listed in the paper. To verify the results' stability, we also analyzed the relationship between tongue fur thickness and tumor marker abnormality in different subgroups divided by gender, age, body mass index (BMI), smoking history, drinking history, tongue color, form of the tongue, and fur color. The analyses were performed with the statistical software packages R (http://www.R-project.org, the *R* Foundation) and EmpowerStats (http://www.empowerstats.com, X&Y Solutions, Inc., Boston, MA). *P* values less than 0.05 (two-sided) were considered statistically significant.

## 3. Results

### 3.1. Baseline Characteristics of Participants

**Figure 2:**
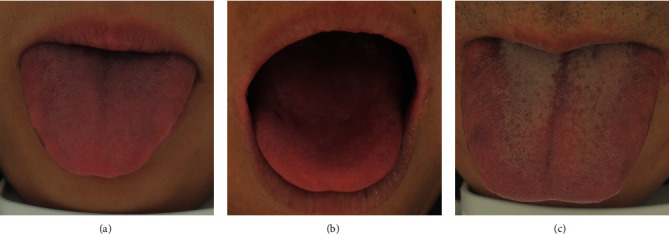
Different tongue fur thickness groups. (a) Thin tongue fur (normal). (b) Less or peeling tongue fur (abnormal). (c) Thick tongue fur (abnormal).

The participants' average age was 48.2 ± 10.4 years old, and about 57.7% were male. The participants were separated into three groups: thin tongue fur, less or peeling tongue fur, and thick tongue fur groups (Figure 2), each having 534, 188, and 903 participants, respectively. The overall rate of tumor marker abnormality in the thin fur group was lower than that in the less or peeling fur group and the thick fur group. Besides, the form of the tongue and the fur color were unevenly distributed in the thin fur, less or peeling fur, and thick fur groups. There was no statistically significant difference in age, gender, BMI, smoking history, drinking history, tongue color, and abnormal rates of t-PSA, AFP, CEA, CA125, and CA199 between the different tongue fur thickness groups (Table 1).

### 3.2. Relationship between Tumor Marker Abnormality and Covariates by Univariable Analysis

Without adjusting any confounding variables, the univariable analysis results showed that (1) compared with the nondrinking group, the risk of having tumor marker abnormality was 1.87 times higher in the drinking group (95% confidence interval: 1.23–2.82, *P*=0.003); (2) compared with the thin tongue fur group, the risk of having tumor marker abnormality was 1.79 times higher in the less or peeling tongue fur group (95% confidence interval: 1.02–3.17, *P*=0.044) and 1.70 times higher in the thick tongue fur group (95% confidence interval: 1.13–2.54, *P*=0.010). The associations between age, gender, smoking history, tongue color, the form of the tongue, fur color, and tumor marker abnormality were not statistically significant (Table 2).

### 3.3. Relationship between Tongue Fur Thickness and Tumor Marker Abnormality by Multivariable Analysis

**Figure 3:**
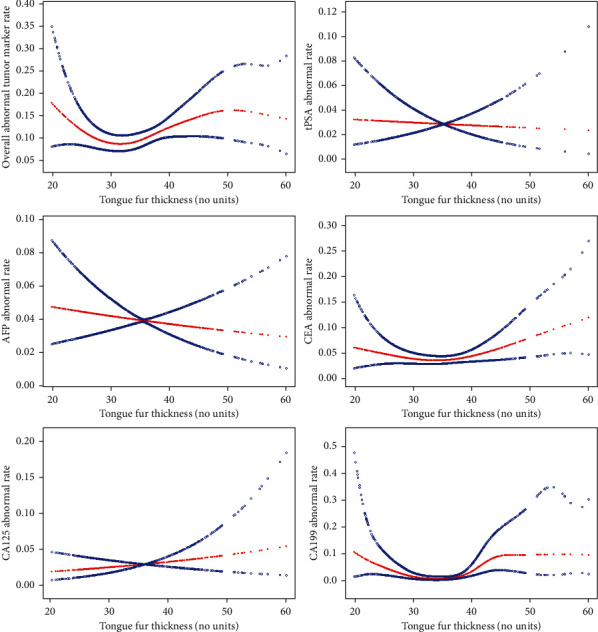
Multivariable-adjusted smoothing spline plots of abnormal tumor marker rate by tongue fur thickness. Red lines represent the spline plots and blue lines represent the 95% confidence intervals (CI) of the spline plots. Adjusted for gender, age, BMI, smoking history, drinking history, tongue color, the form of the tongue, and fur color.

Curve-fitting showed that after adjusting for gender, age, BMI, smoking history, drinking history, tongue color, the form of the tongue, and fur color, the relationship between tongue fur thickness (continuous variable) and overall tumor marker abnormality was nonlinear, like a U shape. As the thickness value of tongue fur increased, the abnormal tumor marker probability first decreased and then increased. We also found this tendency in the relationships between tongue fur thickness and CEA and CA199 abnormalities (Figure 3).

**Figure 4:**
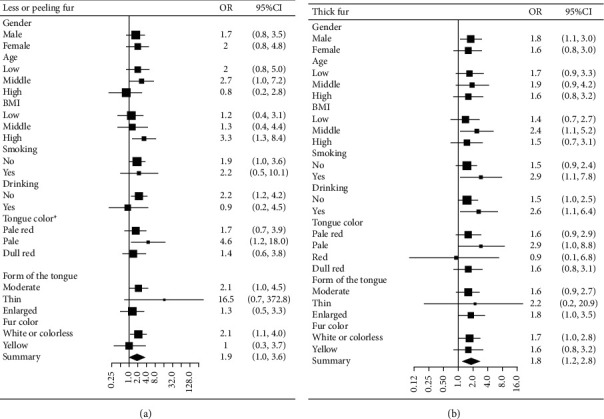
Subgroup analysis. (a) Effect of less fur or peeling fur on abnormal tumor marker probability in different subgroups. (b) Effect of thick fur on abnormal tumor marker probability in different subgroups. ^*∗*^Due to the small sample size, the model failed to calculate the OR value in the red tongue color subgroup.

We subsequently regarded tongue fur thickness as a categorical variable (thin fur, less or peeling fur, and thick fur groups) and set overall tumor marker abnormality as the outcome variable. Multivariable logistic regression showed that after adjusting for gender, age, and drinking history, the odds ratio (OR) values and 95% confidence intervals of the less or peeling fur group and thick fur group for tumor marker abnormality were 1.92 (1.05–3.51) and 1.75 (1.15–2.67), respectively. In the fully adjusted model, we adjusted for gender, age, BMI, smoking history, drinking history, tongue color, the form of the tongue, and fur color. The less or peeling fur group and the thick fur group's OR values showed 1.93 (1.04–3.57) and 1.82 (1.17–2.81), respectively. The results of the univariable and multivariable logistic regression were consistent (Table 3). Subgroup analysis also indicated that for both less or peeling tongue fur and thick tongue fur, the direction of the OR was almost consistent in different subgroups separated by gender, age, BMI, smoking history, drinking history, tongue color, the form of the tongue, and fur color (Figure 4).

When setting each tumor marker abnormality as the outcome variable, multivariable logistic regression did not show statistically significant associations between tongue fur thickness and each tumor marker abnormality (Supplementary table (available here).

## 4. Discussion

For the first time, this study detected a *U*-shaped nonlinear relationship between tongue fur thickness and tumor marker abnormality. Compared with the thin tongue fur group, the less or peeling tongue fur group was 1.9 times more likely to have tumor marker abnormality, and the thick tongue fur group was 1.8 times more likely to have tumor marker abnormality. Univariable and multivariable analysis results were consistent, and OR values were mostly in the same direction (OR > 1) in all subgroups, which further supports the stability of the results.

Epidermal growth factor (EGF) and transforming growth factor-*α* (TGF-*α*) excessive secretion and nutrient deficiency are probable explanations for this nonlinear association. EGF and TGF-*α* are peptides that regulate cell growth [14, 15] and are both thought to be involved in cancer development [16]. Animal experiments have shown that they can promote the proliferation of tongue epithelial cells in mice and rats [17, 18]. We assume that because EGF and TGF-*α* both promote the proliferation of human tongue epithelial cells and tumor cells, it leads to the noncausal association between thick tongue fur and tumor marker abnormality. Likewise, we speculate that the association between less or peeling tongue fur and tumor marker abnormality is noncausal as well. It might be due to the deficiencies of vitamin B6, B12, folic acid, iron, and zinc since they are considered contributing factors of less or peeling tongue fur [19] and can affect the regulation of some oncogenes and increase cancer risk [20–22].

Our findings provide evidence to support the ancient TCM practitioners' understanding of tongue fur thickness. According to the TCM tongue diagnosis theory, the normal thickness of tongue fur should be thin and uniform, while very thick or very thin (or absent) tongue fur may indicate some diseases or prediseases in the body [23]. Correspondingly, modern research has observed that gastroesophageal reflux disease and type 2 diabetes patients tend to have thick tongue fur [24, 25], while primary dysmenorrhea patients tend to have less tongue fur [26]. From the perspective of tumor markers, our results also suggest that people with thick tongue fur or peeling or less tongue fur are more likely to have health issues.

Moreover, since our studied tumor markers (t-PSA, AFP, CEA, CA125, and CA199) are the most commonly used tumor markers in abdominal and pelvic tumors and play an important role in cancer detection and management [27], our study results imply an association between tongue fur thickness and cancer. Previous studies found that, compared with healthy controls, lung, breast, gastric, and colorectal cancer patients have thicker tongue fur [28–31], while peeling or less tongue fur is more prevalent in the lung, gastric, and colorectal cancer patients [28]. These discoveries suggest the potential value of tongue fur thickness for cancer diagnosis and screening. However, prior studies were all case-control designs; therefore, they may be affected by spectrum bias which produces an overestimation of the index test accuracy [32]. Our study design is cross-sectional and can avoid this bias [33]. Based on previous results and ours, it is reasonable to hypothesize that people with very thick or very thin tongue fur are more likely to have cancer. Further cross-sectional studies investigating the relationship between tongue fur thickness and cancer are warranted to confirm this hypothesis and assess the diagnostic accuracy of the tongue fur thickness test.

Lower risk of measurement bias is our study's strength. Previous studies [8–10] evaluated participants' tongue fur thickness by doctors' naked eyes. Since the naked-eye assessment highly depends on the observer's personal experience, there is great potential for variability between different doctors. In contrast, our results measured using the same standardized diagnostic instrument may have better replicability [34]. Besides, the traditional assessing method can only separate tongue fur thickness into three categories (thin, less or peeling, and thick), while modern tongue diagnostic instruments further acquire sophisticated continuous values, which makes it possible to illustrate the nonlinear relationship.

## 5. Limitations

We acknowledge the following four limitations: First, due to the small number of positive results in each tumor marker test, we cannot yet determine which specific tumor marker (t-PSA, AFP, CEA, CA125, or CA199) is associated with tongue fur thickness. Curve-fitting showed a nonlinear tendency in the association of tongue fur thickness and CEA/CA199 abnormality, but the OR values were not statistically significant in multivariable regression models, which needs further studies with a larger sample size to verify this. Second, this is a single-center study without random sampling; therefore, participants in this study may not represent all the general population of China. Third, the relationship of tongue fur thickness and tumor marker abnormality identified in this study is an association rather than a causation; hence, the potential use of tongue fur thickness is limited in the diagnostic and prognostic fields instead of helping to prevent or treat cancer. Finally, a lack of information on residual confounding variables (such as dietary habits and radiation exposure history) prevented us from assessing these variables as potential confounders.

## 6. Conclusions

Tongue fur thickness is nonlinearly associated with tumor marker abnormality. Compared with thin tongue fur people, people with thick tongue fur or peeling or less tongue fur are more likely to have tumor marker abnormality. We hypothesize that very much or very little tongue fur is associated with cancer prevalence, which warrants further cross-sectional studies to confirm and assess the diagnostic accuracy of the tongue fur thickness test for cancer diagnosis and screening.

## Figures and Tables

**Table 1:** Baseline characteristics of 1625 participants according to tongue fur thickness.

Characteristics	Total	Tongue fur thickness			*P* value
		Thin (normal)	Less or peeling	Thick	
Number	1625	534	188	903	

Age (years, mean ± sd)	48.2 ± 10.4	48.0 ± 10.1	47.1 ± 10.3	48.6 ± 10.5	0.224

BMI (kg/m^2^, mean ± sd)	24.5 ± 3.4	24.8 ± 3.4	24.4 ± 3.3	24.4 ± 3.4	0.076

Gender (*n*, %)					0.115
Male	937 (57.7%)	326 (61.0%)	110 (58.5%)	501 (55.5%)	
Female	688 (42.3%)	208 (39.0%)	78 (41.5%)	402 (44.5%)	

Smoking (*n*, %)					0.331
No	1233 (80.6%)	402 (81.5%)	136 (84.0%)	695 (79.4%)	
Yes	297 (19.4%)	91 (18.5%)	26 (16.0%)	180 (20.6%)	

Drinking (*n*, %)					0.590
No	1299 (84.9%)	413 (83.8%)	136 (84.0%)	750 (85.7%)	
Yes	231 (15.1%)	80 (16.2%)	26 (16.0%)	125 (14.3%)	

Tongue color (*n*, %)					0.603
Pale red	598 (36.8%)	201 (37.6%)	72 (38.3%)	325 (36.0%)	
Pale	400 (24.6%)	125 (23.4%)	38 (20.2%)	237 (26.2%)	
Red	28 (1.7%)	10 (1.9%)	2 (1.1%)	16 (1.8%)	
Dull red	599 (36.9%)	198 (37.1%)	76 (40.4%)	325 (36.0%)	

Form of the tongue (*n*, %)					**0.022**
Moderate	790 (48.6%)	270 (50.6%)	85 (45.2%)	435 (48.2%)	
Thin	100 (6.2%)	34 (6.4%)	3 (1.6%)	63 (7.0%)	
Enlarged	735 (45.2%)	230 (43.1%)	100 (53.2%)	405 (44.9%)	

Fur color (*n*, %)					**< 0.001**
White or colorless	1060 (65.2%)	385 (72.1%)	147 (78.2%)	528 (58.5%)	
Yellow	565 (34.8%)	149 (27.9%)	41 (21.8%)	375 (41.5%)	

Tumor marker abnormality (*n*, %)					
Overall	152 (9.4%)	35 (6.6%)	21 (11.4%)	96 (10.6%)	**0.024**
t-PSA	21 (2.5%)	8 (2.8%)	3 (3.0%)	10 (2.2%)	0.838
AFP	51 (3.6%)	14 (3.0%)	9 (5.4%)	28 (3.6%)	0.366
CEA	57 (4.0%)	13 (2.8%)	9 (5.4%)	35 (4.5%)	0.231
CA125	24 (3.4%)	5 (2.4%)	3 (3.8%)	16 (3.7%)	0.666
CA199	29 (2.0%)	5 (1.1%)	5 (3.0%)	19 (2.4%)	0.178

The amount of missing data for the characteristics are as follows: 223 (13.7%) for age, 229 (14.1%) for BMI, 95 (5.8%) for smoking history, and 95 (5.8%) for drinking history. Bold values are considered statistically significant. BMI = body mass index.

**Table 2:** Crude association of abnormal tumor markers with demographic, behavioral, and tongue manifestation characteristics.

Variables	Statistics	Effect size (OR, 95% CI)	*P* value
Gender (*n*, %)			
Male	937 (57.7%)	Reference	
Female	688 (42.3%)	0.88 (0.62, 1.23)	0.453

Age (years, mean ± sd)	48.3 ± 10.4	0.99 (0.98, 1.01)	0.459
Age tertile (*n*, %)			
Low	421 (30.0%)	Reference	
Middle	476 (34.0%)	0.76 (0.50, 1.15)	0.198
High	505 (36.0%)	0.75 (0.50, 1.12)	0.161

BMI (kg/m^2^, mean ± sd)	24.5 ± 3.4	0.95 (0.91, 1.00)	0.070
BMI tertile (*n*, %)			
Low	465 (33.4%)	Reference	
Middle	460 (33.0%)	0.75 (0.50, 1.13)	0.167
High	469 (33.6%)	0.70 (0.46, 1.06)	0.088

Smoking (*n*, %)			
No	1233 (80.6%)	Reference	
Yes	297 (19.4%)	1.32 (0.88, 1.98)	0.182

Drinking (*n*, %)			
No	1299 (84.9%)	Reference	
Yes	231 (15.1%)	1.87 (1.23, 2.82)	**0.003**

Tongue fur thickness			
Thin tongue fur	534 (32.86%)	Reference	
Less or peeling tongue fur	188 (11.57%)	1.79 (1.02, 3.17)	**0.044**
Thick tongue fur	903 (55.57%)	1.70 (1.13, 2.54)	**0.010**

Tongue color (*n*, %)			
Pale red	598 (36.8%)	Reference	
Pale	400 (24.6%)	0.68 (0.43, 1.06)	0.091
Red	28 (1.7%)	1.81 (0.67, 4.94)	0.244
Dull red	599 (36.9%)	0.81 (0.55, 1.19)	0.281

Form of the tongue (*n*, %)			
Moderate	790 (48.6%)	Reference	
Thin	100 (6.2%)	0.52 (0.22, 1.23)	0.137
Enlarged	735 (45.2%)	0.73 (0.51, 1.03)	0.072

Fur color (*n*, %)			
White or colorless	1060 (65.2%)	Reference	
Yellow	565 (34.8%)	1.10 (0.78, 1.56)	0.573

OR = odds ratio, CI = confidence interval, and BMI = body mass index. Bold values are considered statistically significant.

**Table 3:** Association between tongue fur thickness and overall tumor marker abnormality in different models.

	Crude model		Minimally adjusted model		Fully adjusted model	
	OR (95% CI)	*P* value	OR (95% CI)	*P* value	OR (95% CI)	*P* value
Thin fur	Reference		Reference		Reference	
Less fur or peeling fur	1.79 (1.02, 3.17)	0.044	1.92 (1.05, 3.51)	0.035	1.93 (1.04, 3.57)	0.037
Thick fur	1.70 (1.13, 2.54)	0.010	1.75 (1.15, 2.67)	0.009	1.82 (1.17, 2.81)	0.008

Crude model adjusted for none. Minimally adjusted model adjusted for gender, age, and drinking history. Fully adjusted model adjusted for gender, age, BMI, smoking history, drinking history, tongue color, form of the tongue, and fur color. OR = odds ratio and CI = confidence interval.

## Data Availability

All data generated or analyzed during this study are included in the Supplemental Files.

## Abbreviations

TCM: Traditional Chinese medicine
t-PSA: Total prostate-specific antigen
AFP: Alpha-fetoprotein
CEA: Carcinoembryonic antigen
CA125: Carbohydrate antigen 125
CA199: Carbohydrate antigen 199
BMI: Body mass index
OR: Odds ratio
CI: Confidence interval.

## Supplementary Materials

Supplementary Materials Supplemental table: it shows the associations between tongue fur thickness and abnormality of each tumor marker in fully adjusted models. Raw data: it stores the raw data supporting the results of this study. Each row represents an observation (participant) and each column represents a variable. Interpretation of variables: it explains the numerical digits in raw data. For example, X1 means the variable gender, 0 means male, and 1 means female. NA refers to a missing value.
