# Supplementary material for: A Nonlinear Association between Tongue Fur Thickness and Tumor Marker Abnormality: A Cross-Sectional Study
Source: Evid Based Complement Alternat Med. 2021 Nov 30;2021:7909850. doi: 10.1155/2021/7909850 (PMC8651357; doi:10.1155/2021/7909850)
Supplement: Supplementary Materials — Supplemental table: it shows the associations between tongue fur thickness and abnormality of each tumor marker in fully adjusted models. Raw data: it stores the raw data supporting the results of this study. Each row represents an observation (participant) and each column represents a variable. Interpretation of variables: it explains the numerical digits in raw data. For example, X1 means the variable gender, 0 means male, and 1 means female. NA refers to a missing value. [file 7909850.f1.zip › 7909850.f1/Supplementary Table.docx]

**Supplemental table:**

**Associations between tongue fur thickness and abnormality of each tumor marker in fully adjusted models.**

|  | tPSA | | AFP | | CEA | | CA125 | | CA199 | |
| --- | --- | --- | --- | --- | --- | --- | --- | --- | --- | --- |
|  | OR (95%CI) | *P*-value | OR (95%CI) | *P*-value | OR (95%CI) | *P*-value | OR (95%CI) | *P*-value | OR (95%CI) | *P*-value |
| Tongue fur thickness |  |  |  |  |  |  |  |  |  |  |
| Thin fur | Reference |  | Reference |  | Reference |  | Reference |  | Reference |  |
| Less fur or peeling fur | 1.16  (0.29, 4.66) | 0.835 | 1.79  (0.72, 4.48) | 0.212 | 2.03  (0.84, 4.91) | 0.118 | 1.53  (0.24, 9.92) | 0.653 | 2.82  (0.78, 10.14) | 0.113 |
| Thick fur | 0.80  (0.29, 2.17) | 0.658 | 1.17  (0.58, 2.33) | 0.663 | 1.49  (0.76, 2.91) | 0.248 | 2.25  (0.62, 8.12) | 0.217 | 2.06  (0.73, 5.80) | 0.171 |
